# Assessment of perinatal outcomes in intrahepatic cholestasis of pregnancy in relation to transaminase and bile acid levels

**DOI:** 10.1186/s12884-026-09064-7

**Published:** 2026-04-16

**Authors:** Mine Gültekin Çalık, Özge Yücel Çelik, Zuhal Köksal, Aykan Yücel

**Affiliations:** University of Health Sciences, Etlik Zübeyde Hanım Gynecology and Obstetrics Training and Research Hospital, Varlık Mah. Etlik Cad., Yenimahalle, Ankara, Türkiye

**Keywords:** Intrahepatic cholestasis of pregnancy, Bile acids, Alanine aminotransferase, Aspartate aminotransferase, Preterm birth, Perinatal outcomes

## Abstract

**Objective:**

To examine whether maternal peak bile acids (BA) and aminotransferases predict adverse perinatal outcomes, particularly preterm birth, in hospitalized patients with intrahepatic cholestasis of pregnancy (ICP).

**Methods:**

This retrospective cohort study included 179 singleton pregnancies complicated by ICP. Patients were stratified according to peak BA levels (< 40 vs. ≥ 40 µmol/L) and ALT categories (≤ 25, 25–125, > 125 IU/L). The primary outcome was preterm birth (< 37 weeks). Secondary outcomes included fetal distress, meconium-stained amniotic fluid, NICU admission, gestational age at diagnosis and delivery, birth weight, and length of hospitalization. Receiver operating characteristic (ROC) analyses were performed to evaluate the discriminatory ability of laboratory parameters, and multivariable logistic regression analysis was used to identify independent predictors of preterm birth.

**Results:**

Preterm birth occurred in 27.4% of pregnancies (49/179). BA ≥ 40 µmol/L was associated with earlier diagnosis and delivery and longer hospitalization. Increasing ALT categories were associated with earlier diagnosis and delivery, longer hospitalization, and lower birth weight. ALT showed the highest discriminatory performance for predicting preterm birth (AUC 0.70), followed by AST and BA (AUC 0.68 for both). In multivariable analysis, BA ≥ 40 µmol/L and ALT > 125 IU/L remained independently associated with preterm birth.

**Conclusion:**

In hospitalized patients with ICP, elevated bile acid and ALT levels are independently associated with an increased risk of preterm birth. Combined evaluation of bile acids and aminotransferases may improve risk stratification and support individualized surveillance and delivery planning.

## Introduction

Intrahepatic cholestasis of pregnancy (ICP) is the most common pregnancy-specific liver disorder and typically presents with pruritus and elevated serum bile acids and/or aminotransferase levels [1, 2]. Although maternal prognosis is generally favorable, ICP has been associated with an increased risk of adverse perinatal outcomes, including spontaneous or iatrogenic preterm birth, fetal distress, meconium-stained amniotic fluid, neonatal intensive care unit (NICU) admission, and, in severe cases, stillbirth [3–5]. Consequently, accurate risk stratification remains a key component of clinical management in affected pregnancies [4, 5]. Bile acids have been shown to affect placental function, fetal cardiac electrophysiology, and myometrial contractility, which may contribute to adverse fetal outcomes [6–10].

Maternal serum bile acid concentration is widely regarded as the primary biochemical marker of disease severity in ICP. Several studies have demonstrated a graded relationship between increasing bile acid levels and adverse fetal outcomes, particularly at concentrations exceeding 40 µmol/L [11, 12]. Accordingly, bile acid thresholds are frequently used to guide clinical surveillance strategies and timing of delivery [13]. However, clinical practice often reveals discordance between bile acid and aminotransferase levels, raising concerns about whether reliance on bile acids alone adequately reflects overall disease severity [2, 8, 14].

In contrast to bile acids, the prognostic significance of aminotransferase elevations—especially alanine aminotransferase (ALT)—has received comparatively less attention. Although transaminase levels may reflect hepatocellular injury and inflammatory activity, their independent contribution to adverse perinatal outcomes in ICP remains controversial [2, 8]. Previous studies investigating aminotransferases as prognostic markers have reported inconsistent findings and frequently included heterogeneous patient populations, often without distinguishing between outpatient and hospitalized cases [14–16].

Hospitalized patients with ICP may represent a clinically distinct subgroup characterized by more severe symptoms, closer maternal and fetal surveillance, and a higher likelihood of obstetric intervention [17, 18]. While bile acids are widely used for risk stratification in intrahepatic cholestasis of pregnancy, the independent prognostic value of aminotransferases—particularly in hospitalized patients with clinically significant disease—remains insufficiently defined. Clarifying this relationship may improve risk assessment and support more individualized clinical decision-making in this higher-risk population.

Therefore, the aim of this study was to evaluate the association between maternal serum bile acid and aminotransferase levels and adverse perinatal outcomes in a cohort of hospitalized patients with intrahepatic cholestasis of pregnancy, with a particular focus on preterm birth and related clinical parameters.

## Materials and methods

This retrospective cohort study was conducted at Etlik Zübeyde Hanım Gynecology and Obstetrics Training and Research Hospital, a tertiary referral center located in Ankara, Türkiye. Medical records of pregnant patients hospitalized with a diagnosis of intrahepatic cholestasis of pregnancy (ICP) between January 2014 and December 2018 were reviewed.

Only hospitalized patients were included in order to reflect a clinically significant ICP population requiring close maternal and fetal monitoring. In our institution, hospitalization is reserved for patients with moderate-to-severe symptoms, markedly elevated biochemical parameters, or increased concern for adverse perinatal outcomes. This approach allowed the evaluation of laboratory parameters in a higher-risk and more homogeneous clinical cohort. This selection strategy intentionally focused on a clinically higher-risk population; therefore, the findings should be interpreted within the context of inpatient ICP management rather than the broader spectrum of mild outpatient disease.

Singleton pregnancies diagnosed with intrahepatic cholestasis of pregnancy (ICP) with available serum bile acid and liver transaminase measurements, and with complete obstetric and neonatal outcome data, were eligible for inclusion. Patients with missing laboratory measurements were not included in the analysis. Patients with multiple gestations, known chronic liver disease, viral hepatitis, preeclampsia-related liver dysfunction, or incomplete medical records were excluded from the analysis.

Peak serum bile acid (BA) levels measured during hospitalization were used for patient stratification, as peak values are considered to better reflect disease severity. Patients were categorized into two groups according to BA levels: <40 µmol/L and ≥ 40 µmol/L, consistent with previously reported thresholds associated with increased perinatal risk.

The ≥ 40 µmol/L threshold was selected a priori as a commonly used clinical severity cut-point in the ICP literature and in delivery-planning algorithms. In addition to this predefined stratification, we performed ROC analyses to explore cohort-specific laboratory cut-offs for discriminating preterm birth; these data-driven cut-offs are presented as exploratory and are not intended to replace guideline-based thresholds. Alanine aminotransferase (ALT) levels were categorized as ≤ 25 IU/L (approximately corresponding to the upper limit of normal in pregnancy), 25–125 IU/L (mild-to-moderate elevation), and > 125 IU/L (marked elevation), reflecting clinically meaningful strata of hepatocellular injury frequently used in prior ICP cohorts. This categorization was chosen to distinguish between normal, moderate, and substantial biochemical elevation while preserving adequate group sizes for analysis. Aspartate aminotransferase (AST) levels were recorded and analyzed as continuous variables in exploratory analyses. All patients received standard clinical management according to institutional protocols. Ursodeoxycholic acid (UDCA) was routinely prescribed for symptomatic relief and biochemical improvement in patients with ICP. During hospitalization, fetal surveillance was performed according to the institutional protocol, typically including regular non-stress testing and clinical assessment, with adjustments based on the patient’s clinical condition. Throughout the study period (2014–2018), the institutional approach to delivery timing and labor induction in patients with ICP remained generally consistent and was based on overall clinical assessment, including gestational age, biochemical severity, and fetal surveillance findings. During the study period, the institutional approach to delivery timing and labor induction in patients with ICP remained generally consistent and was based on overall clinical assessment, including gestational age, biochemical severity, and fetal surveillance findings. In our institution, UDCA is typically initiated at a dose of 10–15 mg/kg/day, divided into two or three doses, and adjusted according to clinical response and biochemical parameters [1, 4, 5]. Due to the retrospective nature of the study and variability in treatment initiation timing, dosing, and duration, treatment-related variables could not be reliably standardized or included in the statistical models. However, because UDCA use was common across the hospitalized cohort, its confounding effect is likely limited, although residual confounding cannot be excluded. The primary outcome was preterm birth, defined as delivery before 37 completed weeks of gestation. Due to the retrospective design and limitations of the medical record system, it was not possible to reliably classify preterm births as spontaneous preterm labor, PPROM, or medically indicated delivery. Therefore, preterm birth was evaluated as a composite outcome including both spontaneous and medically indicated (iatrogenic) deliveries. Secondary outcomes included fetal distress, meconium-stained amniotic fluid, neonatal intensive care unit (NICU) admission, gestational age at diagnosis and delivery, birth weight, and duration of hospitalization.

### Statistical analysis

Continuous variables were assessed for normality using visual inspection and appropriate tests and were summarized as mean ± standard deviation or median (minimum–maximum), as appropriate. Categorical variables were expressed as number and percentage. Comparisons between two groups were performed using the Mann–Whitney U test for continuous variables and the chi-square or Fisher’s exact test for categorical variables. Comparisons among more than two groups were conducted using the Kruskal–Wallis test. Receiver operating characteristic (ROC) curve analyses were performed to assess the ability of bile acid, ALT, and AST levels to discriminate preterm birth and to explore potential laboratory cut-off values. Optimal cut-off values were identified using the Youden index and should be interpreted as exploratory; predefined thresholds (e.g., BA ≥ 40 µmol/L) were used for clinical severity stratification. To identify independent predictors of preterm birth, a multivariable logistic regression model was constructed. Variables were selected a priori based on clinical relevance and biological plausibility. The model included bile acid category (≥ 40 vs. < 40 µmol/L), ALT category (> 125 vs. ≤ 125 IU/L), and gestational age at diagnosis, which is a known determinant of delivery timing in ICP. Results were reported as adjusted odds ratios (aORs) with 95% confidence intervals (CIs). A two-sided p-value < 0.05 was considered statistically significant. Statistical analyses were performed using IBM SPSS Statistics for Windows, Version 17.0 (IBM Corp., Armonk, NY, USA).

## Results

A total of 179 hospitalized patients with intrahepatic cholestasis of pregnancy were included in the analysis. The mean maternal age was 27.0 ± 5.0 years and the mean body mass index (BMI) was 28.6 ± 5.0 kg/m². The median gestational age at diagnosis was 34 weeks (range: 22–40). The mean serum bile acid level was 32.4 ± 35.1 µmol/L, while the mean ALT and AST levels were 121.1 ± 123.7 IU/L and 75.0 ± 69.3 IU/L, respectively. Baseline maternal demographic and clinical characteristics of the study population are summarized in (Table 1). The overall cesarean delivery rate in the study cohort st frequency observed in patients with ALT > 125 IU/L.

**Table 1:** Maternal demographic and clinical characteristics of the overall cohort

Variable	Overall cohort (*n* = 179)
Age (years), mean ± SD	27.0 ± 5.0
Body mass index (kg/m²), mean ± SD	28.6 ± 5.0
Gestational week at diagnosis, median (min–max)	34 (22–40)
Duration of hospitalization (days), median (min–max)	8 (2–34)
ALT (IU/L), mean ± SD	121.1 ± 123.7
AST (IU/L), mean ± SD	75.0 ± 69.3
Serum bile acids (µmol/L), mean ± SD	32.4 ± 35.1
Birth weight (g), mean ± SD	3001.6 ± 551.2
APGAR score (1st minute), median (min–max)	9 (0–9)
APGAR score (5th minute), median (min–max)	10 (3–10)
Systemic disease, n (%)	17 (9.5)
Previous history of ICP, n (%)	5 (2.8)
Gravidity (nulligravida), n (%)	83 (46.4)
Parity (nulliparous), n (%)	95 (53.1)
Gestational diabetes, n (%)	10 (5.6)
Mode of delivery (cesarean), n (%)	92 (51.4)
Non-stress test (non-reactive), n (%)	3 (1.7)

In our cohort, preterm birth occurred in 49 of 179 pregnancies (27.4%). Among these cases, 31 (63.3%) were classified as spontaneous preterm births and 18 (36.7%) as medically indicated (iatrogenic) deliveries. Due to limitations of the retrospective medical record system, spontaneous preterm labor and preterm premature rupture of membranes (PPROM) could not be reliably distinguished and were therefore analyzed together as spontaneous preterm birth.

When examined according to ALT categories, spontaneous preterm births occurred in 3 patients with ALT ≤ 25 IU/L, 14 patients with ALT 25–125 IU/L, and 14 patients with ALT > 125 IU/L, while medically indicated preterm deliveries occurred in 1, 4, and 13 patients in these groups, respectively. These findings suggest that medically indicated preterm delivery was more frequent in patients with higher ALT levels.

When stratified by peak serum bile acid concentration, patients with BA ≥ 40 µmol/L were diagnosed at an earlier gestational age and delivered earlier compared with those with BA < 40 µmol/L. In addition, the duration of hospitalization was significantly longer in the BA ≥ 40 µmol/L group (Table 2). Rates of fetal distress and meconium-stained amniotic fluid did not differ significantly between groups; however, preterm birth and NICU admission occurred more frequently among patients with BA ≥ 40 µmol/L. No cases of stillbirth were observed in the study cohort during the study period. However, stillbirth is a relatively rare outcome in ICP, and larger studies are required to evaluate this association better. Because the present cohort reflects clinical practice between 2014 and 2018, admission decisions were based on overall clinical assessment rather than bile acid levels alone. Therefore, some patients with bile acid levels < 40 µmol/L were hospitalized for closer maternal and fetal monitoring.

**Table 2:** Perinatal outcomes according to bile acid level (< 40 vs. ≥ 40 µmol/L)

Variable	BA < 40 µmol/L (*n* = 140)	BA ≥ 40 µmol/L (*n* = 39)	*p* value
Gestational week at diagnosis, median (min–max)	34.7 (22.7–40.1)	32.9 (22.3–39.0)	0.033
Gestational week at delivery, median (min–max)	37.5 (36.8–41.0)	37.0 (30.6–40.0)	0.010
Duration of hospitalization (days), median (min–max)	7 (2–34)	10 (3–32)	0.016
APGAR score (1st minute), median (min–max)	9 (5–9)	9 (0–9)	0.688
APGAR score (5th minute), median (min–max)	10 (8–10)	10 (3–10)	0.664
Preterm birth, n (%)	32 (22.9)	17 (43.6)	0.010
Fetal distress, n (%)	13 (9.3)	3 (7.7)	1.000
Meconium-stained amniotic fluid, n (%)	5 (3.6)	3 (7.7)	0.371
NICU admission, n (%)	12 (8.6)	13 (33.3)	< 0.001

Because a large proportion of the study population was overweight or obese, an additional logistic regression analysis was performed, adjusting for maternal BMI. Maternal BMI was not independently associated with preterm birth (*p* = 0.917) or NICU admission (*p* = 0.652). The association between higher bile acid levels and preterm birth remained significant after adjustment for BMI (*p* = 0.004).

Increasing ALT categories were associated with progressively earlier gestational age at diagnosis and delivery, longer hospitalization, higher bile acid levels, and lower birth weight (Table 3). Preterm birth rates increased significantly across ALT strata, with the highest frequency observed in patients with ALT > 125 IU/L. In the present cohort, 103 patients had bile acid levels below 40 µmol/L but elevated ALT levels (> 25 IU/L). Among these patients, 28 (27.2%) delivered preterm, suggesting that aminotransferase elevation may also reflect increased perinatal risk even when bile acid levels remain below conventional severity thresholds.

**Table 3:** Perinatal outcomes according to ALT category (≤ 25, 25–125, > 125 IU/L)

Variable	ALT ≤ 25 (*n* = 39)	ALT 25–125 (*n* = 85)	ALT > 125 (*n* = 55)	*p* value
Gestational week at diagnosis, mean ± SD	36 ± 4	34 ± 3	32 ± 4	< 0.01
Gestational week at delivery, mean ± SD	38 ± 1	37 ± 2	36 ± 3	< 0.01
Serum bile acids (µmol/L), mean ± SD	19.5 ± 18.1	36.0 ± 37.0	36.1 ± 39.3	< 0.01
Duration of hospitalization (days), mean ± SD	5.7 ± 3.0	9.2 ± 5.2	11.3 ± 7.1	< 0.01
Birth weight (g), mean ± SD	3183 ± 464	2980 ± 567	2908 ± 564	0.045
APGAR score (1st minute), mean ± SD	8.7 ± 0.7	8.7 ± 0.8	8.5 ± 1.3	0.47
APGAR score (5th minute), mean ± SD	9.8 ± 0.4	9.7 ± 0.6	9.6 ± 1.0	0.06
Preterm birth, n (%)	4 (11.1)	18 (24.0)	27 (51.9)	< 0.01
NICU admission, n (%)	3 (8.3)	12 (16.0)	10 (19.2)	0.40
Meconium-stained amniotic fluid, n (%)	2 (5.6)	2 (2.7)	4 (7.7)	0.50

**Fig. 1:**
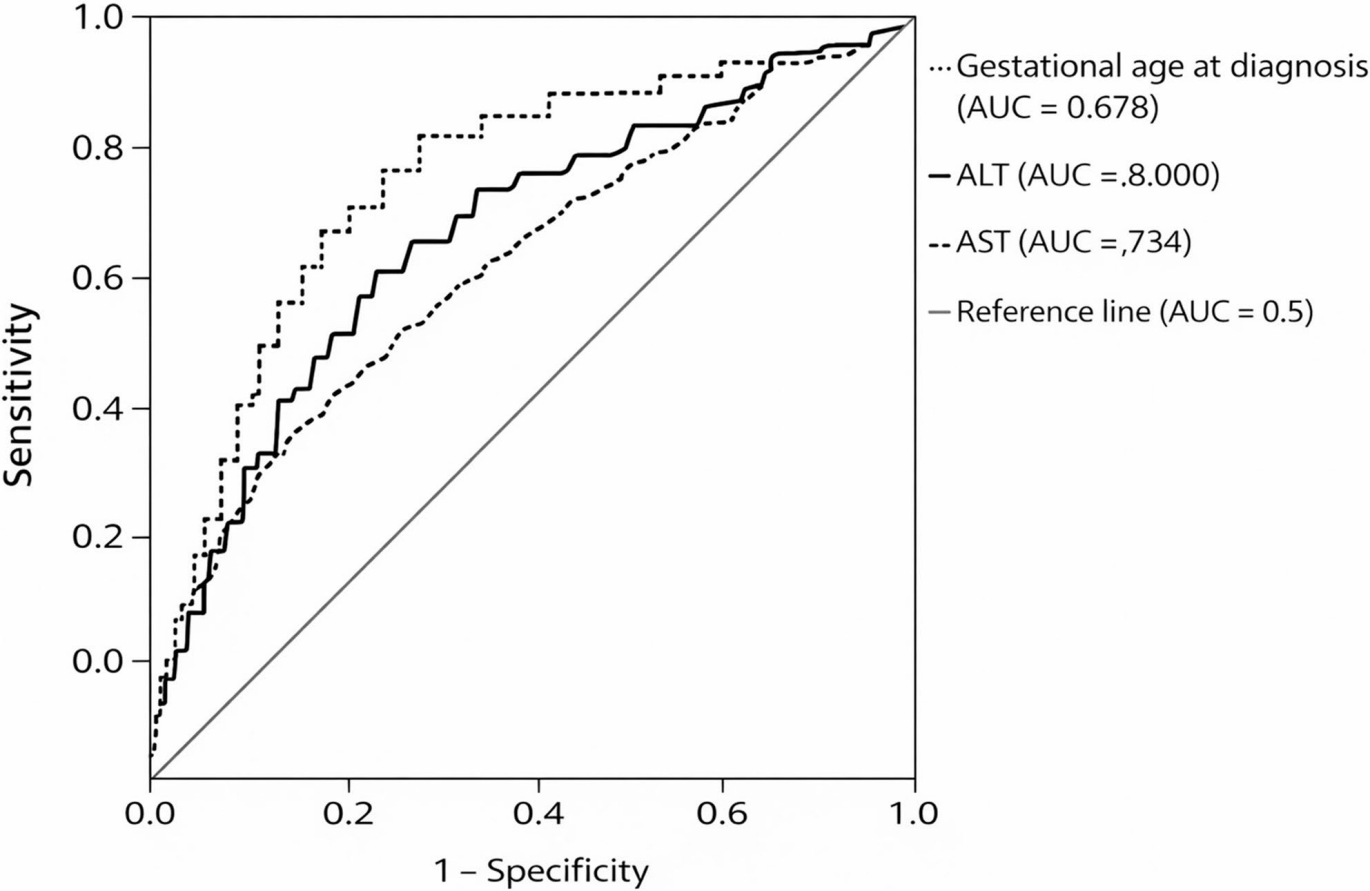
Receiver operating characteristic (ROC) curves of bile acid, ALT, and AST levels for the prediction of preterm birth

Receiver operating characteristic analyses demonstrated modest discriminatory ability of ALT, AST, and bile acid levels for preterm birth, with ALT showing the highest area under the curve (AUC 0.70). Cut-off values and corresponding sensitivity and specificity are presented in (Table 4). ROC curves for bile acid, ALT, and AST levels predicting preterm birth are presented in (Fig. 1). The ROC-derived BA cut-off (19.25 µmol/L) reflects the cohort-specific point of best sensitivity/specificity trade-off and therefore differs from the predefined ≥ 40 µmol/L severity threshold used for stratified analyses.

**Table 4:** ROC analysis for prediction of preterm birth

Variable	AUC	*p* value	Cut-off	Sensitivity (%)	Specificity (%)
ALT	0.70	< 0.05	99.5	67	66
AST	0.68	< 0.05	73	59	74
Serum bile acid	0.68	< 0.05	19.25	76	60

In multivariable logistic regression analysis, bile acid levels ≥ 40 µmol/L and ALT levels > 125 IU/L remained independently associated with preterm birth after adjustment for gestational age at diagnosis.

## Discussion

In this retrospective cohort of hospitalized patients with intrahepatic cholestasis of pregnancy (ICP), higher maternal bile acid and aminotransferase levels were associated with adverse perinatal outcomes, particularly those related to preterm birth. Consistent with previous studies, elevated bile acid levels were linked to earlier diagnosis, earlier delivery, and prolonged hospitalization, supporting the role of bile acids as markers of disease severity and clinical decision-making in ICP [2, 11]. Several large cohort studies and meta-analyses have demonstrated a graded relationship between increasing bile acid concentrations and adverse fetal outcomes, including fetal distress, meconium-stained amniotic fluid, and preterm birth [3, 11, 12]. In particular, individual patient data analyses have shown a marked increase in fetal complications at bile acid levels ≥ 40 µmol/L [11]. Our findings are consistent with this evidence, as patients with bile acid levels ≥ 40 µmol/L exhibited earlier delivery and longer inpatient management. Because the present cohort reflects clinical practice between 2014 and 2018, admission decisions were based on overall clinical assessment rather than bile acid levels alone. Therefore, some patients with bile acid levels < 40 µmol/L were hospitalized for closer maternal and fetal monitoring. In the present cohort, a considerable proportion of patients were overweight or obese. Because maternal BMI may influence pregnancy outcomes, additional analyses were performed adjusting for BMI. In these analyses, maternal BMI was not independently associated with preterm birth or NICU admission. Importantly, the association between higher bile acid levels and preterm birth remained significant after adjustment for BMI. These findings suggest that the relationship between elevated bile acid levels and adverse perinatal outcomes in ICP is unlikely to be explained solely by maternal BMI. We used a bile acid threshold of ≥ 40 µmol/L for group stratification because this level is widely recognized as a marker of more severe ICP and is frequently used to guide surveillance intensity and timing of delivery [2, 18]. In contrast, ROC analysis in our cohort yielded a lower bile acid cut-off (19.25 µmol/L) for the prediction of preterm birth. This ROC-derived threshold should be interpreted as an exploratory cohort-specific estimate rather than a replacement for the clinically established 40 µmol/L threshold, particularly given the modest discriminatory performance observed. The lower threshold may partly reflect the clinical management of hospitalized high-risk patients, in whom earlier delivery decisions may be influenced by biochemical severity [2, 19]. Beyond bile acids, aminotransferase levels—particularly ALT—provided additional prognostic information in our cohort. Higher ALT categories were associated with earlier diagnosis and delivery, longer hospitalization, higher bile acid levels, and lower birth weight. Previous studies have suggested that elevations in liver transaminases may reflect hepatocellular involvement and disease severity in ICP [14, 15]. Ekiz et al. also reported that ALT elevation may predict adverse perinatal outcomes independently of bile acid levels, supporting the potential value of aminotransferases in risk assessment [8]. Receiver operating characteristic analyses in our study demonstrated only modest discrimination of bile acids and aminotransferases for predicting preterm birth. This finding is consistent with contemporary evidence indicating that risk prediction in ICP is limited when based on a single laboratory parameter and that the risk of adverse outcomes increases along a continuum of disease severity rather than at a single universal cut-off value [7, 11, 20, 21]. Despite the modest discriminatory performance observed in the ROC analysis, these findings may still provide clinically relevant information by highlighting the potential contribution of aminotransferase levels, particularly ALT, to risk stratification in patients with ICP. Therefore, biochemical markers should be interpreted together with clinical findings and fetal surveillance results when assessing perinatal risk in patients with ICP. These observations highlight the importance of integrating biochemical markers with clinical features such as gestational age at diagnosis, symptom burden, and fetal surveillance findings, as emphasized in recent guideline-based risk stratification strategies [1, 2]. Importantly, in our multivariable analysis, bile acid levels ≥ 40 µmol/L and ALT levels > 125 IU/L remained independently associated with preterm birth after adjustment for gestational age at diagnosis. The independent association of ALT > 125 IU/L suggests that aminotransferase elevation may reflect additional aspects of disease activity not fully captured by bile acid concentrations alone. Taken together, these findings indicate that combined assessment of bile acids and aminotransferases may help identify higher-risk patients more effectively than reliance on a single biochemical marker. The relatively high cesarean delivery rate in our cohort may reflect the tertiary referral nature of our center and the tendency toward closer fetal surveillance and earlier obstetric intervention in pregnancies complicated by ICP. In hospitalized patients with moderate-to-severe ICP, biochemical severity and fetal surveillance findings can influence decisions regarding the timing of delivery. Therefore, the association between laboratory markers and preterm birth in our cohort may partly reflect both underlying disease severity and obstetric management decisions. The observed association between higher bile acid or aminotransferase levels and preterm birth in our cohort may partly reflect both underlying fetal risk and obstetric intervention, a phenomenon commonly referred to as confounding by indication. Future studies should attempt to distinguish spontaneous preterm birth from medically indicated early delivery and evaluate whether biochemical markers improve the prediction of spontaneous prematurity beyond clinical risk factors.

Several limitations should be acknowledged. First, the retrospective design limits causal inference and is subject to potential information bias. Because of the retrospective design and the structure of the hospital record system, the exact number of initially screened cases and excluded patients could not be reliably determined. Second, the study population consisted exclusively of hospitalized patients, who may represent a subgroup with more severe clinical or biochemical disease; therefore, the findings may not be fully generalizable to women with milder ICP managed in the outpatient setting. Third, treatment-related factors such as ursodeoxycholic acid use and timing of delivery—known to influence outcomes in ICP—could not be fully controlled [4, 18]. Fourth, although the overall number of spontaneous and medically indicated preterm births could be determined, the medical records did not allow reliable differentiation between spontaneous preterm labor and preterm premature rupture of membranes (PPROM). In addition, the specific obstetric indications leading to medically indicated preterm delivery could not be consistently determined from the medical records, which limited further analysis of the underlying causes of early delivery.

## Conclusion

Higher bile acid (≥ 40 µmol/L) and aminotransferase levels (particularly ALT > 125 IU/L) are associated with worse perinatal outcomes among hospitalized patients with ICP. These laboratory strata may help identify patients who warrant closer monitoring and individualized delivery planning. 

## Data Availability

The data that support the findings of this study are available from the corresponding author upon reasonable request.
